# Seroconversion of Hepatitis B Vaccine in Young Children in the Kassena Nankana District of Ghana: A Cross-Sectional Study

**DOI:** 10.1371/journal.pone.0145209

**Published:** 2015-12-30

**Authors:** Sylvester Dassah, Samuel A. Sakyi, Margaret T. Frempong, Arnold T. Luuse, Richard K. D. Ephraim, Enoch O. Anto, Abraham Oduro

**Affiliations:** 1 Department of Molecular Medicine, School of Medical Sciences, Kwame Nkrumah University of Science and Technology (KNUST), Kumasi, Ghana; 2 Navrongo Health Research Centre, Navrongo, Upper East, Ghana; 3 Faculty of Allied Health Science, University of Health and Allied Sciences, Ho, Ghana; 4 Department of Medical Laboratory Technology, University of Cape Coast, Cape Coast, Ghana; Tulane University, UNITED STATES

## Abstract

**Background:**

Hepatitis B Virus (HBV) infection is an important public health problem that requires high priority efforts towards prevention and control. Active immunization is the single most important and effective preventive measure against HBV infection. As a protective measure, Ghana introduced the mass immunization program against hepatitis B infection in children in 2002 in her Expanded Programme on Immunization (EPI). This study evaluated seroconversion (the point in time when the amount of antibody in the blood becomes detectable) and seroprotection (the point in time when the amount of antibody in the blood is enough to confer protection from the antigen that induced it production) status of children under this mass immunization program and measured their antibody levels five years after immunization.

**Materials and Method:**

200 archived plasma samples of children between the ages of 1–10 years were retrieved from a previous cross-sectional study by researchers from NHRC between 2009 and 2010. Of these, 104 have completed the EPI and were screened for HBsAg. Those found to be HBsAg-seronegative were stratified into three groups according to their age at which the last vaccine was administered. Their anti-HBsAg titer levels were estimated by enzyme linked immunosorbant assay (ELISA).

**Results:**

Two (1.9%) samples were HBsAg seropositive and were excluded from further analyses. 10 more samples were excluded from analyses because they were insufficient. The anti-HBs titers recorded ranged from 1.021 IU/L to 751.64 IU/L indicating a 100% seroconversion rate. In group one (0–6 months), 87.9% were seroprotected. Group two (2-3yrs) had 78.3% seroprotection and group three (3-5yrs) had 41.7% seroprotection. There was no significant difference between group 1 and 2. However, there was a significant difference between group 1 and 3 (*p* = 0.0137) and between group 2 and 3 (*p* = 0.0390) respectively. There was no significant difference between male and female children.

**Conclusion:**

All the children who received doses of hepatitis B vaccine at 6, 10 and 14 weeks in the immunization program seroconverted, but their levels of protection waned with increasing years. Booster doses are therefore recommended after 5 years.

## Introduction

The World Health Organization (WHO) has estimated that two billion people (one-third of the world’s population) have serologic evidence of past or present hepatitis B virus (HBV) infection and 360 million are chronic carriers who are at risk of liver disease [1]. Approximately, 620,000 deaths occur every year as a result of secondary complications to hepatitis B and 4.5 million new cases of hepatitis B are reported each year worldwide [2]. Chronic hepatitis B has been identified as one of the most common causes of liver failure and hepatocellular carcinoma. Many preventive measures have been employed, including screening of blood donors, preparation of plasma-derived products in a way that inactivates hepatitis B virus, the implementation of infection control measures, and administration of hepatitis B immunoglobulin [3]. However, active immunization with hepatitis B vaccine remains the single most important hepatitis B prevention measure. Following the availability of hepatitis B vaccines for the prevention of HBV infection in 1982, the vaccine has been used in different parts of the world. Significant impact has been observed on the incidence of HBV infection through the implementation of mass immunization programs among infants, children, and adolescents in many countries [2,4].

Populations in the intermediate and high endemic regions are more at risk of acquiring HBV infection if not vaccinated [5]. Sero-prevalence surveys, indicates that Ghana falls within the endemic regions, with HBV carrier rates between 2.2% to 13.8% in some districts [6,7]. The WHO recommended in 1991 that hepatitis B vaccination should be included in national immunization programs for all countries with hepatitis B carrier prevalence of 8% or greater by 1995 and in all other countries by 1997. Ghana started mass immunization program on hepatitis B in infants in 2002 with a single-combined EPI vaccine; pentavalent vaccine (DTPw–HepB–Hib; Panacea Biotech ltd, India). This vaccine are administered at weeks 6, 10 and 14 respectively.

It is anticipated that this standard schedule of immunization should produce 95% seroprotection rate as revealed in international data [4]. However, factors such as genetic make-up, immunosuppression, vaccine storage conditions, obesity, diabetes, and gender have been implicated to adversely affect the immune response to hepatitis B vaccination [8,9]. Moreover, there is paucity of data regarding seroconversion as well as seroprotection among the recipients of hepatitis B vaccine in Ghana since its implementation in 2002. It is against this background that this study sought to evaluate the seroconversion and seroprotection status of children who have received the hepatitis B vaccine, determine their antibody levels 5 years post vaccination under the EPI program in Ghana, and finally to ascertain the success rate of the vaccination program to help future policy decision making.

## Materials and Methods

### Ethical considerations

The study protocol was approved by the Institutional Review Board (IRB) of Navrongo Health Research Center (NHRC), and the Committee on Human Research Publication and Ethics (CHRPE) of the School of Medical Sciences, Kwame Nkrumah University of Science and Technology. Written informed consent were sought from parents and guardians of children whose archived samples were used in the study. This was done during a follow up outreach program in the district.

### Study Area

The study was conducted at the Navrongo Health Research Center (NHRC) located in the Kassena Nankana District in the Upper East Region of Ghana. The War Memorial Hospital in Navrongo is a referral center and serves as the recruitment point for most of the biomedical studies conducted by the NHRC. The District Health Directorate carried out immunization exercises at clinics, outreaches in the communities and house-to-house immunizations through home visits and supplementary immunization activities.

### Study population and subject selection

**Fig 1:**
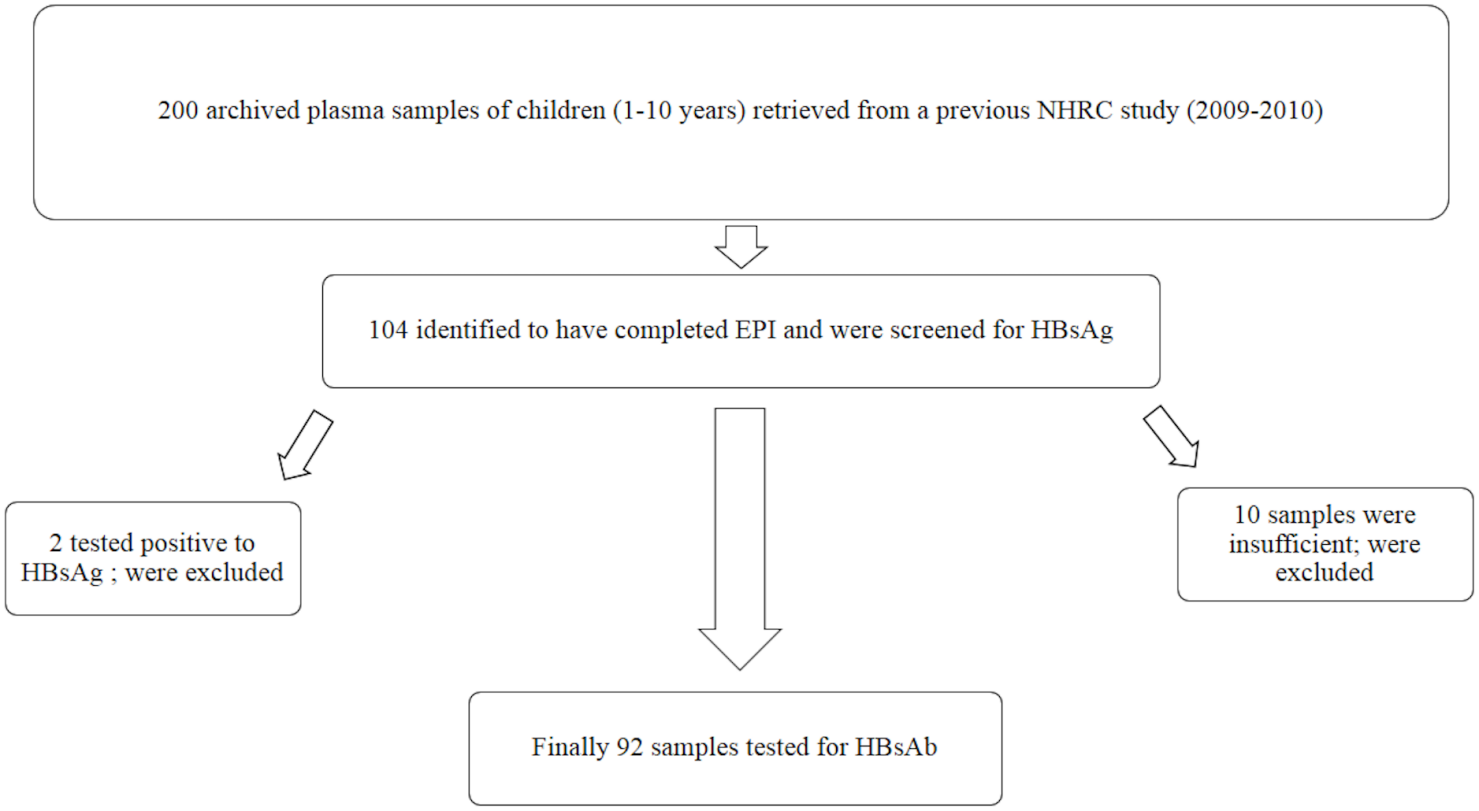
Flow chart showing study population and subject selection.

Two hundred (200) plasma samples of children between the ages of 1–10 years were retrieved from a previous cross-sectional study conducted between 2009 and 2010 by researchers from NHRC. The samples had been collected from healthy children within the community during an outreach program, and were being stored at -80°C in the research laboratory at NHRC. The samples were previously used for the determination of sero-prevalence of antibodies against candidate malaria vaccine viral vectors, EPI programs and for establishment of normal laboratory reference values. The samples were checked against available records, 104 samples were identified to have completed the EPI vaccination program and were selected as subjects for this study. The selected 104 samples were stratified according to the following categories: Group 1 (1 to 6 months after their last dose of vaccination), Group 2 (3 years after their last dose of vaccination) and, Group 3 (5 years after their last dose of vaccination). The vaccination records of participants were obtained from the Navrongo Demographic Surveillance System (NDSS) and verified from the vaccination cards with 100% accuracy. Fig 1 shows how the study population and subjects were selected.

### Sample analysis

The 104 samples identified to have completed the EPI were screened for HBsAg by immunochromatographic test strip assay kit (Guangzhou Wondfo Biotech Co., Ltd., China). Out of these, 2 were HBsAg seropositive and were excluded from further analyses. 10 more samples were excluded because they were not sufficient for analysis. The remaining 92 HBsAg negative samples were included in the quantification of antibody to hepatitis B virus surface antigen (anti-HBs). The presence of antibodies produced against the recombinant antigen were determined by ELISA using the Wantei Hep.BV kit (Beijing Wantai Biological Pharm. Enterprise CO., Ltd, China). Each test was performed in duplicates according to the manufacturer’s instructions, the plate was read on ELISA plate reader Emax (Molecular Devices LLC, USA). All the sample analysis were carried out in the research laboratory of Navrongo Health Research Center.

### Data Analyses

Data was entered into an Excel spread sheet. OD’s from the ELISA plate reader including standards were converted into actual concentrations using an Auditable Data Analysis and Management System for ELISA (ADAMSEL-V1.1). All data analyses were performed using STATA and *p* ≤ 0.05 was considered statistically significant. Anti-HBs titers were converted into log titers before one-way ANOVA followed by Tukey Post Hoc multiple comparison was used to test for significance. Fischer exact test was used to compare the differences in degree of seroprotection between the males and females.

Individuals with antibody concentration of ≥1mIU/mL were considered to have seroconverted and vice visa as per kit manufacture instructions and WHO standard. Seroprotection against hepatitis B virus infection was taken to be antibody titer ≥ 10mIU/ml.

## Results

### General characteristics

Forty-five (45) out of the final 92 samples were male children whereas 47 were females. The average weight of all the children was 13.19kg (lower-upper weight: 6.8–21.3kg) and their mean age was 3.12years (lower-upper age: 0.11–6.50years). A review of the vaccination records of the children showed that none of them had had a post-exposure prophylaxis or dose at birth. They, however, had complete records for all the three scheduled doses at 6, 10 and 14 weeks.

### Seroconversion status of hepatitis B vaccinees

All the 92 children that were included in the quantification of anti-HBs seroconverted (i.e. had anti-HBs ≥1 mIU/mL). Anti-HBs titers recorded ranged from 1.021 IU/L to 751.64 IU/L. The geometric mean titer (GMT) for group one antibody was 96.12 IU/L (range: 1.02 IU/L to 573.64 IU/L). Among group two, the GMT was 83.78 IU/L (range: 2.15 IU/L to 225.76 IU/L). In group three, the GMT was 37.55 IU/L (range: 2.39 IU/L to 751.64 IU/L). Among males, the GMT was 66.67 IU/L whilst 79.90 IU/L was recorded among the females (Tables 1 and 2).

**Table 1:** Antibody Concentration among the three age Groups.

Mean titer (IU/L) of Groups (95% CI)			
Group 1	Group 2	Group 3	p-value
96.12* (63.24–146.10)	83.78a ^‡^ (53.58–130.99)	37.55a* (18.82–74.93)	0.02

One-way ANOVA followed by Tukey Post Hoc multiple comparison. Values with same superscript (*) (Group 1 vs 3) and subscript (a) (Group 2 vs 3) are significantly different compared to each other whilst values with different superscript (‡) (Group 1 vs 2) are not statistically different compared to each other.

**Table 2:** Antibody concentration among male and female vaccine.

Age groups	Mean titer (IU/L) (95% CI) of males	Mean titer (IU/L) (95% CI) of females
Group 1	83.7(47.65–147.02)	110.38 (55.36–220.08)
Group 2	103.43 (48.83–219.07)	71.85 (37.70–137.02)
Group 3	25.6 (13.47–48.67)	52.51 (14.29–192.92)
All Groups	66.66 (45.10–98.57)	79.9 (51.81–123.22)

### Anti-HBs titer and their association with protection

Seroprotection analyses (Table 3) revealed that in group one, 87.9% were seroprotected after 1 to 6 months of vaccination and 12.1% were non-responders. In group two, 78.3% were seroprotected after 3 years of completing vaccination schedule while 21.7% were non-responders. In group three, 41.7% were seroprotected after 5 years of completing vaccination schedule whereas 58.3% were non-responders. This trend indicates that seroprotection declines as the vaccinees get older though this was not statistically significant (Table 1) between age group one and two (p > 0.05); however, there was a significant difference between age group one and three (p = 0.0137) and between age group two and three (p = 0.0390).

**Table 3:** Seroprotection among the vaccinees in the three age groups.

	Anti-HBs titre (IU/L)		
		Responder	
Participants	Non-responder	Hypo-responder	Good responder
	(< 10 IU/L)	(10–100 IU/L)	(>100 IU/L)
Group 1(n = 33)	4(12.12%)	13(39.39%)	16(48.48%)
Group 2(n = 23)	5(21.74%)	11(47.83%)	7(30.43%)
Group 3(n = 36)	21(58.33%)	12(33.33%)	3(8.33%)
Overall	30 (32.61%)	62 (67.40)	

### The effect of age and sex on the immune response to hepatitis B vaccine

Table 4 indicates the effect of age and sex on immune response to HBV infection. 93.3% of males and 83.3% of females in group one were seroprotected (anti-HBs titre ≥ 10 IU/L) in group two, 88.9% of the males and 71.4% of the females were seroprotected whilst in group three 33.3% of males and 53.3% of the females were seroprotected. In all these groups there were differences in degree of seroprotection between the males and females but these differences were not statistically significant (p > 0.05).

**Table 4:** Age group and sex among the vaccinees in relation to seroprotection.

Age groups/Sex	Non-responders: Anti-HBs titre	Responders: Anti-HBs titre	p value
	(< 10 IU/L)	(> 10 IU/L)	
Group 1:			
Male (15)	6.67% (n = 1)	93.33% (n = 14)	
Female (18)	16.67 (n = 3)	83.33% (n = 15)	0.508
Group 2:			
Male (9)	11.11% (n = 1)	88.89% (n = 8)	
Female (14)	28.57% (n = 4)	71.43% (n = 10)	0.414
Group 3:			
Male (21)	66.67% (n = 14)	33.33% (n = 7)	
Female (15)	46.67% (n = 7)	53.33% (n = 8)	0.282
Overall:			
Male (45)	35.56% (n = 16)	64.44% (n = 29)	
Female (47)	29.79% (n14)	70.21% (n = 33)	0.533

### Distribution of Antibody concentration among vaccinees within the three age groups

**Fig 2:**
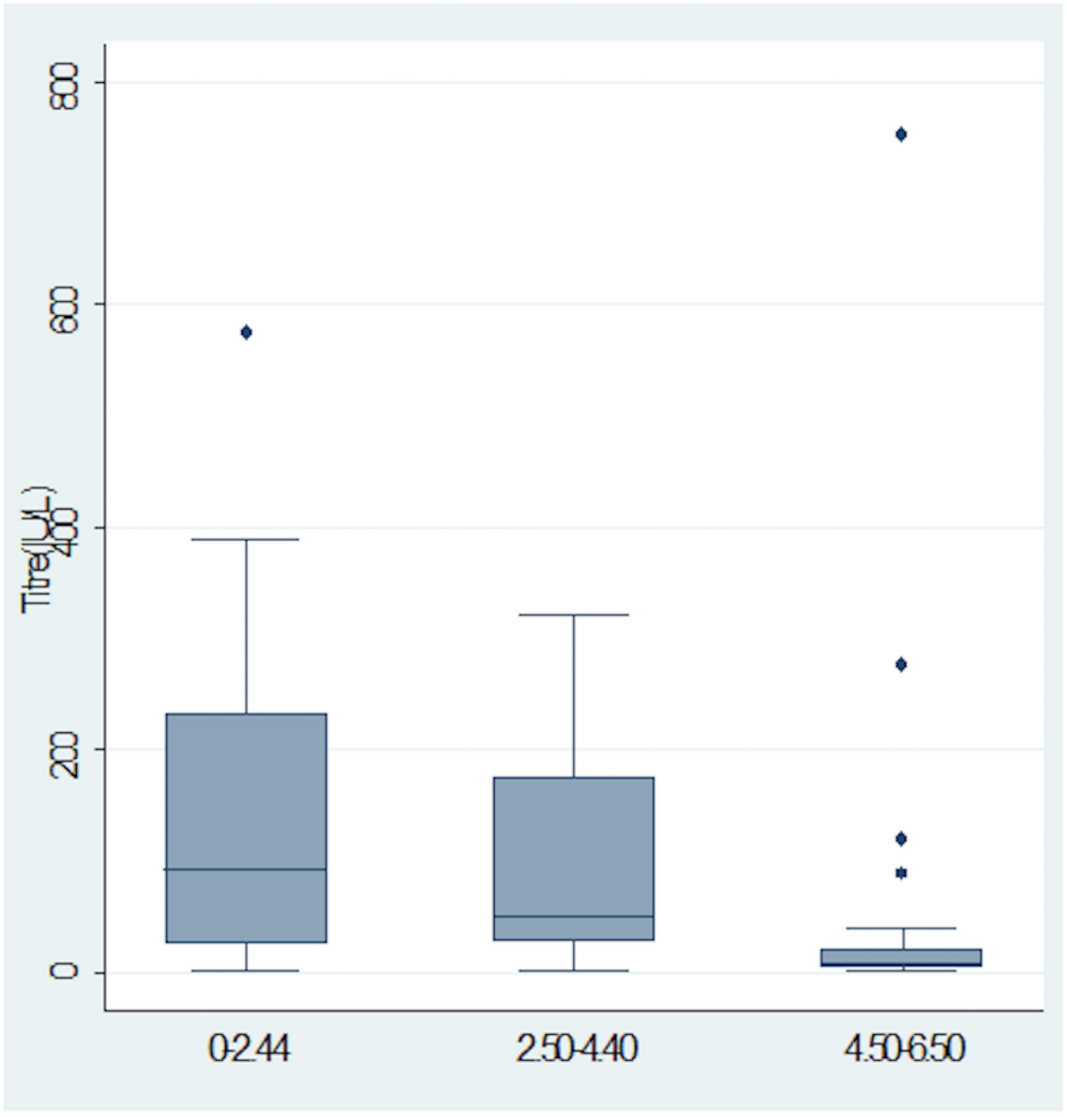
Box-plot showing the distribution of Antibody concentration among vaccinees within the three age groups. Figure is represented as median (interquartile range).

Median antibody concentration were observed to decrease with increasing age (p<0.05) as indicated in Fig 2.

### Distribution of Antibody concentration among males and female vaccinees within the three age groups

**Fig 3:**
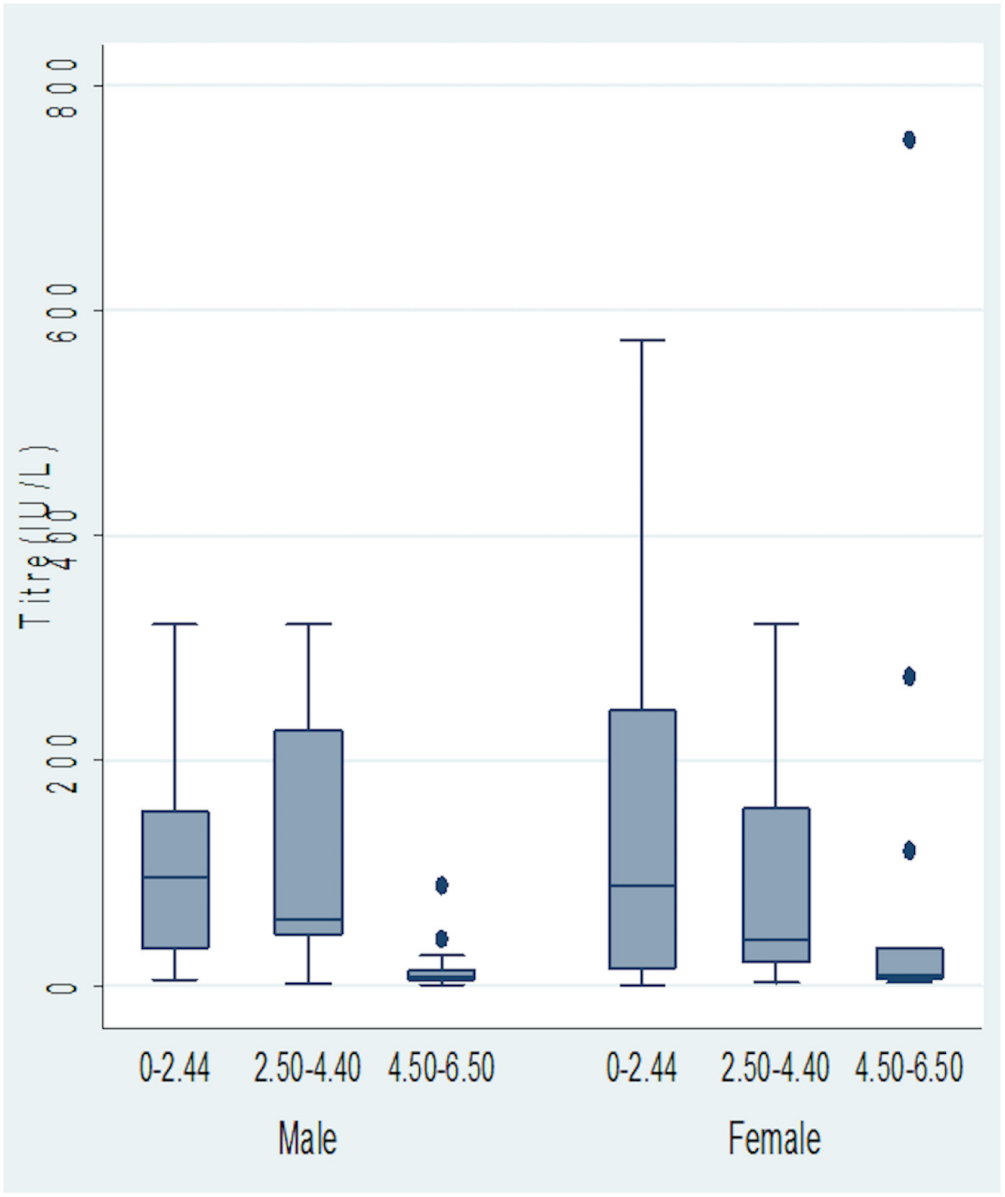
Box-plot showing the distribution of Antibody concentration among males and female vaccinees within the three age groups. Figure is represented as median (interquartile range)

**Fig 4:**
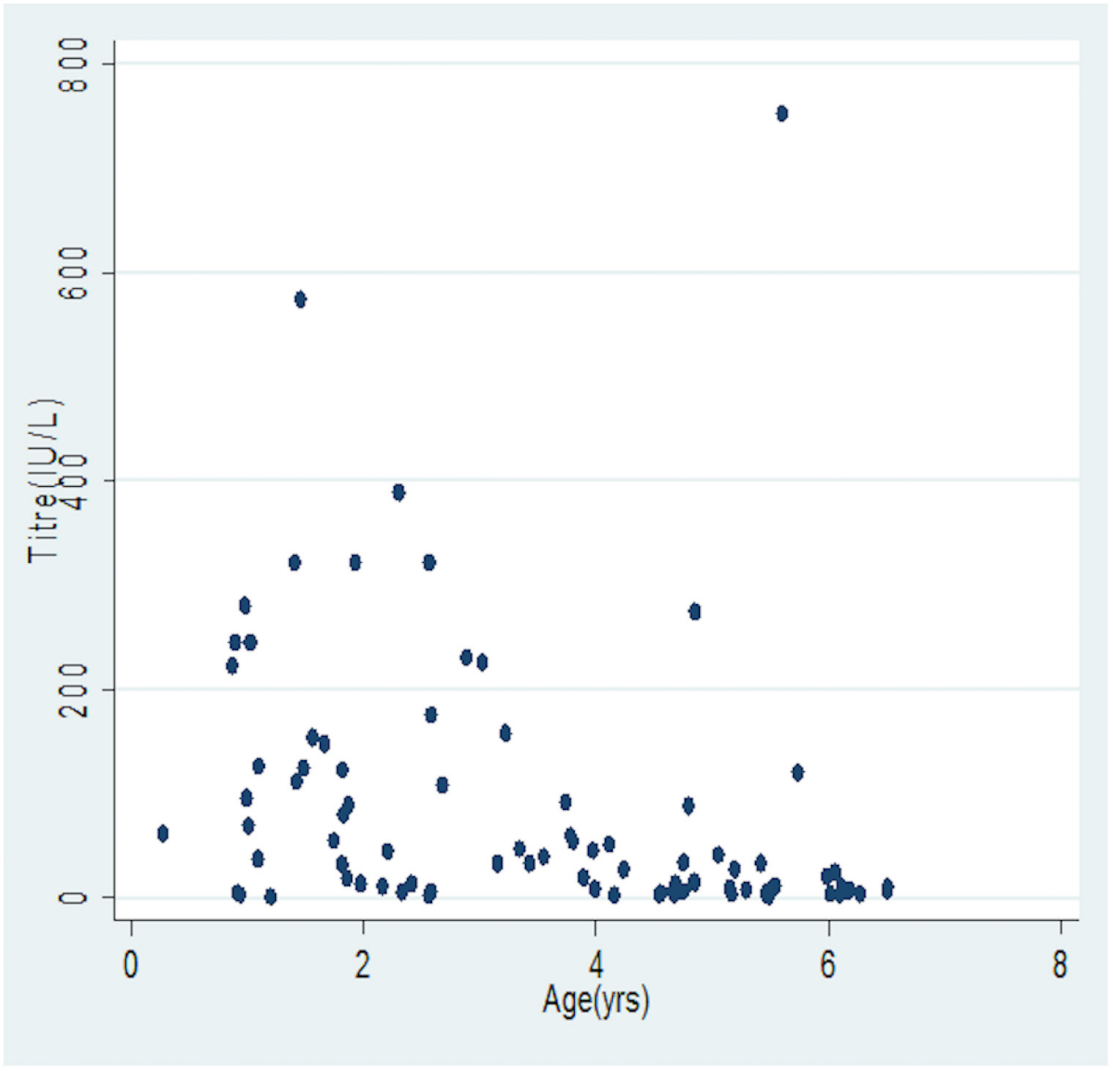
Scatter plot of antibody concentration with age.

In Fig 3, the median antibody concentrations were observed to decrease with increasing age irrespective of gender (p<0.05), while Fig 4 shows variations of antibody concentration with age.

## Discussion

This study evaluated seroconversion and seroprotection status of children under HBV mass immunization program and measured their antibody levels five years after immunization. All the children in the study showed levels of seroconversion (i.e. had anti-HBs ≥ 1 IU/L) after three doses of hepatitis B vaccination in the EPI. Some of the antibody levels however, did not reach concentrations that confers protection. Seroprotection levels indicated a waning protective immunity in the amounts of anti-HBs produced by the children from 87.9% to 41.7%. Furthermore, this study showed a varying rate of non-protection (12.1% after 1 to 6 months, 21.7% after 3 years and 58.3% after 5 years). This study further revealed that, 1 to 6 months after receiving three doses of hepatitis B vaccine in the EPI, 12.1% of the population of remain vulnerable to HBV infection. When extrapolated into our vaccination coverage it indicates that a large number of children remain exposed to risk of HBV infection. There was no gender differences in the immunological response to hepatitis B vaccine among our study group.

Findings from our study is consistent with a longitudinal studies by Freitas da Motta *et al*., [10] in Brazil, who reported, 98% of individuals seroconverted. Moreover, Chakraborty *et al*., [11] reported 100% seroconversion in a cross-sectional study in Bangladesh. In two separate studies conducted in South Africa and Brazil, 86.80% and 90.00% seroprotection levels were reported [12–14]. Guho and colleagues [15] in Bangladesh also reported 88.67% of seroprotection in their study. The waning immunity observed in our study is consistent with findings by El-Sayed *et al*., [16] who reported that anti-HB titers decline over time. In a long-term study to evaluate protection of hepatitis B vaccination among children immunized when infants, Dentinger and colleagues [17] also found that anti-HBs concentration dropped rapidly among all participants. Moreover, findings from other studies also showed a varying rate of non-protection. Puvacic *et al*., [18] reported that vaccination against viral hepatitis B results in immunologic memory response among the vaccinated and that even after a decrease of anti-HB levels to below the protective level of ≥ 10 IU/L, a booster dose was not needed. Lu *et al*., [19] on the other hand reported 29.2% non-responsiveness to a booster dose by vaccinees that initially seroconverted with non-protective levels. The need for booster dose for long term protection into adolescence and adulthood need to be well investigated and a consensus reached on the large pool of non-responders being reported across the globe. In contrast to our findings, Behairy *et al*., [16] reported that males retain higher anti-HBsAb titres values than females. [20]. The findings of this current study is consistent with the report of some studies which had reported that there was no gender differences in the immunological response to hepatitis B vaccine [21,22].

The main limitation of the current study is that archived samples were used and this impacted on the sample size. Nevertheless, the findings from this study corresponded well with previous studies and is adequate to represent the baseline information about seroconversion and seroprotection under the EPI program.

## Conclusion

All the children who received three doses of hepatitis B vaccine at 6, 10 and 14 weeks seroconverted, but their levels of protection waned with increasing years. Booster doses are therefore recommended after 5 years for long term protection into adolescence and adulthood.

This study will serve as a baseline for further studies with prospective cohort design involving large sample size to establish the strength of this observation.
